# High level in vivo mucin-type glycosylation in *Escherichia coli*

**DOI:** 10.1186/s12934-018-1013-9

**Published:** 2018-10-26

**Authors:** Phillipp Mueller, Rahul Gauttam, Nadja Raab, René Handrick, Claudia Wahl, Sebastian Leptihn, Michael Zorn, Michaela Kussmaul, Marianne Scheffold, Bernhard Eikmanns, Lothar Elling, Sabine Gaisser

**Affiliations:** 1 Institute of Applied Biotechnology, Biberach University of Applied Sciences, Biberach, Germany; 2 Institute of Microbiology and Biotechnology, University of Ulm, Ulm, Germany; 3 Institute for Biotechnology and Helmholtz-Institute for Biomedical Engineering, RWTH Aachen University, Aachen, Germany; 4 Zhejiang University-Edinburgh University Institute, School of Medicine, Zhejiang University, Zhejiang, China; 5 Boehringer Ingelheim Pharma GmbH and Co.KG, Analytical Development Biologics, Biberach, Germany

**Keywords:** Protein expression, *Escherichia coli*, Glycosyltransferase GalNAcT2, Chaperone co-expression, In vivo glycosylation, UDP-GlcNAc 4-epimerase WbgU, Mucin-type *O*-glycosylation

## Abstract

**Background:**

Increasing efforts have been made to assess the potential of *Escherichia coli* strains for the production of complex recombinant proteins. Since a considerable part of therapeutic proteins are glycoproteins, the lack of the post-translational attachment of sugar moieties in standard *E. coli* expression strains represents a major caveat, thus limiting the use of *E. coli* based cell factories. The establishment of an *E. coli* expression system capable of protein glycosylation could potentially facilitate the production of therapeutics with a putative concomitant reduction of production costs.

**Results:**

The previously established *E. coli* strain expressing the soluble form of the functional human-derived glycosyltransferase polypeptide *N*-acetylgalactosaminyltransferase 2 (GalNAc-T2) was further modified by co-expressing the UDP-GlcNAc 4-epimerase WbgU derived from *Plesiomonas shigelloides*. This enables the conversion of uridine 5′-diphospho-*N*-acetylglucosamine (UDP-GlcNAc) to the sugar donor uridine 5′-diphospho-*N*-acetylgalactosamine (UDP-GalNAc) in the bacterial cytoplasm. Initially, the codon-optimised gene *wbgU* was inserted into a pET-derived vector and a Tobacco Etch Virus (TEV) protease cleavable polyhistidine-tag was translationally fused to the C- terminus of the amino acid sequence. The 4-epimerase was subsequently expressed and purified. Following the removal of the polyhistidine-tag, WbgU was analysed by circular dichroism spectroscopy to determine folding state and thermal transitions of the protein. The in vitro activity of WbgU was validated by employing a modified glycosyltransferase assay. The conversion of UDP-GlcNAc to UDP-GalNAc was shown by capillary electrophoresis analysis. Using a previously established chaperone pre-/co- expression platform, the in vivo activity of both glycosyltransferase GalNAc-T2 and 4-epimerase WbgU was assessed in *E. coli*, in combination with a mucin 10-derived target protein. Monitoring glycosylation by liquid chromatography electrospray ionization mass spectrometry (LC–ESI–MS), the results clearly indicated the in vivo glycosylation of the mucin-derived acceptor peptide.

**Conclusion:**

In the present work, the previously established *E. coli*- based expression system was further optimized and the potential for in vivo *O*-glycosylation was shown by demonstrating the transfer of sugar moieties to a mucin-derived acceptor protein. The results offer the possibility to assess the practical use of the described expression platform for in vivo glycosylations of important biopharmaceutical compounds in *E. coli*.

**Electronic supplementary material:**

The online version of this article (10.1186/s12934-018-1013-9) contains supplementary material, which is available to authorized users.

## Background

Since the first approval of the recombinantly expressed human therapeutic protein Humulin^®^ in 1982, *Escherichia coli* has served as reliable and cost-efficient production host in the pharmaceutical industry [1]. In recent years the requirements of modern biopharmaceuticals have shifted towards higher complexity, while consistently maintaining high product quality [2]. Over the years, the lack or insufficient capability of *E. coli* strains to perform post-translational modifications, including disulfide bond formation and glycosylations [2, 3], has contributed to the rise of mammalian rather than non-mammalian expression systems for the commercial production of biopharmaceutical products [4]. However, as a very popular expression host, *E. coli* remains an important factor in biopharmaceutical manufacturing [4]. To enable recombinant expression of complex proteins in *E. coli*, research has focused on improving protein folding, disulfide bond formation, as well as *N*- and *O*-linked glycosylation [5–11]. *N*-linked glycosylation in *E. coli* has been achieved by transferring the *pgl* gene cluster derived from *Campylobacter jejuni* into *E. coli* with the subsequent expression of glycosyltransferases and enzymes required for sugar biosynthesis in the strain background [10–12]. The glycan is assembled on a membrane-anchored precursor molecule and flipped into the periplasm, where the oligosaccharyltransferase PglB attaches the glycan onto the target protein [12]. However, the glycosylation site consensus sequence of PglB is more stringent than the eukaryotic polypeptide acceptor sequence. As a result, engineering of the polypeptide sequence of the target protein or altering the substrate specificity of PglB is required to promote correct *N*-glycosylation of recombinant proteins expressed in the periplasm of *E. coli* [13]. In contrast, *O*-glycosylation of eukaryotic target sites using a truncated form of the human glycosyltransferase GalNAc-T2 has been shown without altering the target peptide sequence [6, 9, 14, 15].

*O*-glycosylation involves the transfer of sugar moieties to a serine or threonine residue, commonly found on mucins. Mucins are cell surface or secreted high-molecular weight glycoproteins on the mucosal epithelium in animals and humans, which provide a protective shield against toxins and pathogens [3, 16]. *O*-glycosylation of mucins is initiated by the transfer of GalNAc—a reaction catalyzed by UDP-*N*-Acetylgalactosaminyltransferases (GalNAc-Ts)—forming the GalNAcα1-*O*-serine or threonine linkage [16]. Different mucin-derived target peptides have been successfully used as substrates to assess the activity of the human glycosyltransferase GalNAc-T2 lacking the N-terminal transmembrane domain [6, 9, 14, 15]. As an example, glycosylation of the synthetic peptide EA2 (PTTDSTTPAPTTK)—derived from *Rattus norvegicus* submandibular apomucin 10 (MUC10)—has been demonstrated in various in vitro assays in the presence of soluble GalNAc-T2 [6, 14, 15].

The in vivo *O*-glycosylation of a glutathione S-transferase-mucin peptide fusion in an *E. coli* SHuffle^®^ T7 express strain co-expressing an N-terminal truncated version of GalNAc-T2, in combination with the UDP-GlcNAc 4-epimerase WbpP from *Pseudomonas aeruginosa* has been indicated based on Western blot analysis using horseradish peroxidase-conjugated *Vicia villosa* lectin [9]. However, the detailed analysis of the glycosylated target mucin peptide with eight potential glycosylation sites has not been shown [9]. Additionally, potential *O*-glycosylation sites comprising eight amino acids of relevant biopharmaceuticals have been fused to anti-TNF-α Fab and analysed for the presence of GalNAcα1-*O*-serine or threonine [9].

As a first step to establish an *E. coli*-based expression platform enabling the glycosylation of full-length biopharmaceuticals in vivo, an active human GalNAcT2 derivative comprising amino acids 52–571 with a translationally fused N-terminal HisDap (diamino peptidase) tag has been isolated from a SHuffle^®^ T7 *E. coli* strain pre-/co-expressing chaperones [6]. The activity of the purified glycosyltransferase has been demonstrated using the mucin peptide derivative EA2 and filgrastim (Granulocyte-colony stimulating factor, G-CSF) as acceptor substrates in vitro [6]. As a further step to develop an *E. coli* expression strain for the production of glycosylated biopharmaceuticals, the presented approach describes the addition of the active *Plesiomonas shigelloides*-derived UDP-GlcNAc 4-epimerase (WbgU), to ensure the presence of the sugar substrate UDP-GalNAc in the *E. coli* strain background. WbgU was chosen based on the well-characterized properties of the enzyme [17]. An artificial protein derived from MUC10 (GenBank: AAA20966.1, UniProtID: Q62605) was used as the target protein. In the present work, we successfully show in vivo glycosylation by co-expressing the 4-epimerase WbgU, the truncated form of the glycosyltransferase GalNAc-T2, and the MUC10 target protein in the established *E. coli* system pre-/co-expressing the chaperones sulfhydryl oxidase Erv1p (essential for respiration and vegetative growth protein 1) and the human protein disulfide isomerase PDI, as previously described [6, 8]. The presence of functional GalNAc-T2, WbgU and soluble expressed MUC10 derivative was demonstrated by immunoblot analysis. WbgU was detected with polyclonal antibodies in serum samples taken from rabbits immunized with purified protein. The detailed analysis of the in vivo glycosylated target protein is shown.

## Results

### Expression, purification, and analysis of WbgU

**Fig. 1:**
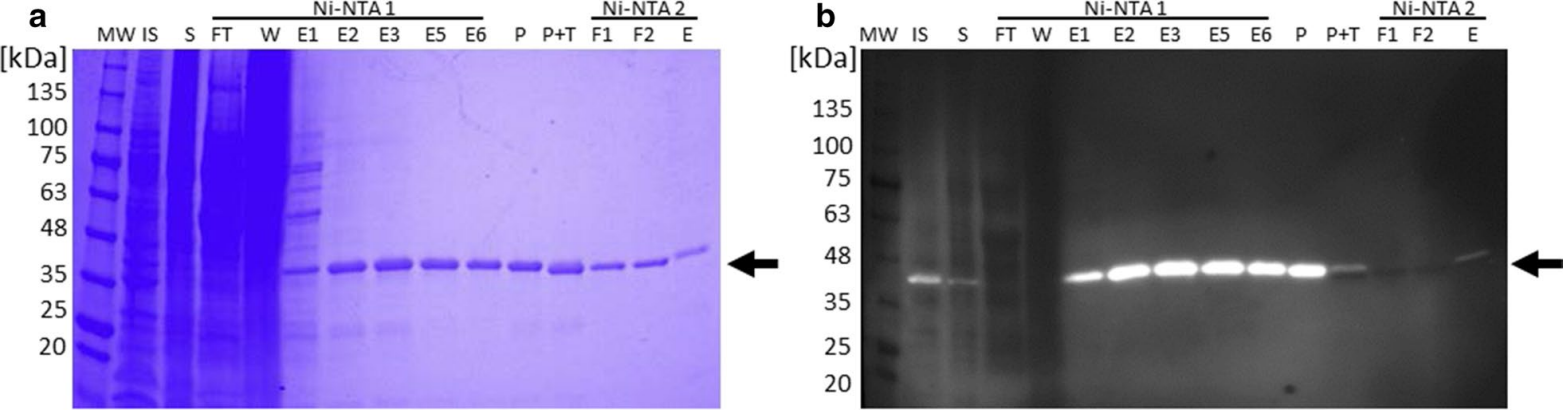
SDS-PAGE (**a**) and immunoblot analysis (**b**) of WbgUTEV6H expression in *E. coli* and purification of WbgU. *E. coli* SHuffle^®^ T7 Express cells carrying plasmids pET23a(+)_*wbgUTEV6H* and pMJS9 were grown in TB medium at 30 °C until OD_600_ of 1 was reached. l-arabinose was added to a final concentration of 0.5% and the culture was incubated for another 6.5 h. The cells were harvested by centrifugation, lysed, and insoluble particulate fractions (IS) and soluble fractions (S) were isolated. WbgUTEV6H was purified from the soluble fraction using Ni–NTA chromatography (Ni–NTA 1). Fractions of the flow-through (FT), washing (W), and elution (E1–E6) were collected. Fractions E2–E6 were pooled (P) and incubated with TEV protease (P + T). The cleaved His-tag, TEV protease, and undigested WbgUTEV6H were removed using a second affinity chromatography step (Ni–NTA 2, E). Aliquots (10 μl) of the samples and collected fractions were separated by SDS-PAGE and visualized by Coomassie staining (**a**) and immunoblotting (**b**). WbgUTEV6H with an estimated mass of 41 kDa was detected using mouse Anti-His.H8 antibodies. Tag-free WbgU (40 kDa) was clearly visible in the flow-through (**a**, F1 and F2). The respective bands were no longer detected in the immunoblot assay with Anti-His.H8 antibodies (**b**, F1 and F2) indicating the successful removal of the polyhistidine tag. Molecular mass markers (MW) are in kDa

Experiments to express the 4-epimerase were performed using *E. coli* host strain SHuffle^®^T7 Express harbouring plasmids pMJS9 and pET23a(+)_*wbgUTEV6H* as previously described [6, 8]. The specific cleavage sequence for the Tobacco Etch Virus nuclear-inclusion-a endopeptidase (TEV protease) inserted between the WbgU amino acid sequence and translationally fused C-terminal polyhistidine tag (6H, His-tag) was used to obtain tag-free enzyme via affinity chromatography and subsequent protease treatment (Fig. 1a, b). Soluble expression of WbgUTEV6H (41 kDa) was found to be best in Terrific Broth (TB) medium without Isopropyl-β-d-thiogalactopyranosid (IPTG) induction and with the pre-/co-expression of the redox folding helper proteins Erv1p and PDI encoded by pMJS9 under the control of an arabinose promotor (data not shown). Soluble WbgUTEV6H (Fig. 1b, lane S) was purified by employing Ni–NTA affinity chromatography. The protein was clearly visible in eluted fractions (Fig. 1a, b, lanes E2–E6), which were pooled and treated with polyhistidine-tagged TEV protease. The cleaved His-tag and TEV protease were removed in a subsequent affinity chromatography step and WbgU identified by N-terminal Edman degradation. The tag-free protein migrated at 40 kDa under reducing denaturing conditions in the SDS-gel and was no longer detected using Anti-His antibodies (Fig. 1a, b, lanes F1 and F2). The protein was further used for the immunization of rabbits to generate polyclonal antibodies for the detection of WbgU in immunoblot assays. The obtained sera samples were assessed and immunoblot conditions were optimized to minimize unspecific signals (data not shown).

### Circular dichroism (CD) spectrum analysis

**Fig. 2:**
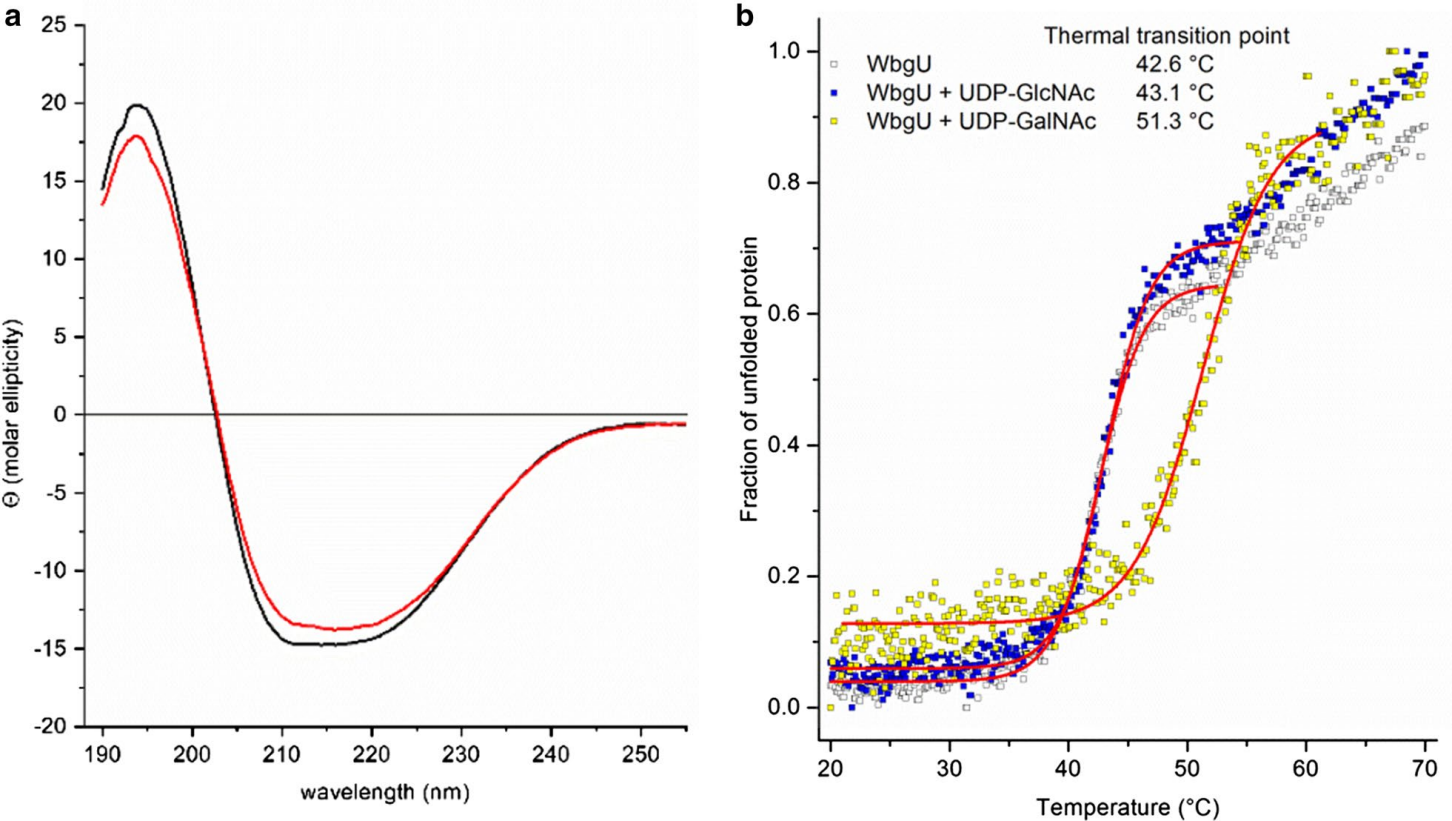
CD spectroscopy and thermal stability measurements of WbgU. **a** Far UV (195–260 nm) spectra of WbgU at 20 °C (black line) and 37 °C (red line) indicating the folding status of the 4-epimerase in 20 mM sodium phosphate buffer at pH 7.75. CD data were reported as molar ellipticity (Θ). **b** Thermal unfolding profiles of WbgU (white squares), WbgU in the presence of either UDP-GlcNAc (blue squares) or UDP-GalNAc (yellow squares) were monitored at 220 nm

The secondary structure and thermal transition points of WbgU were assessed by CD spectroscopy (Fig. 2). Characteristic minima at 208 and 222 nm, typical for α-helical structures, were identified in CD scans indicating the folding of WbgU (Fig. 2a). The slight difference in the scans measured at 20 °C and 37 °C potentially indicates some reduction of secondary structures at the higher temperature. However, it has already been shown by Kowal and Wang, that WbgU exhibits the highest activity at 37 °C [17]. The thermal unfolding profiles of WbgU were monitored by recording temperature-dependent molar ellipticity changes at 220 nm from 20 °C to 70 °C. The thermal transition points at 42.55 ± 0.04 °C, increasing to 43.07 ± 0.04 °C, and 51.29 ± 0.13 °C in the presence of UDP-GlcNAc and UDP-GalNAc, respectively, were determined based on the sigmoid graphs displayed in Fig. 2b. We concluded that the increase detected in samples containing UDP-GalNAc may indicate a stabilizing effect in the presence of the sugar substrate. Similarly, an enhancement of the conformational stability has been postulated for the human UDP-galactose 4′-epimerase GALE when binding the substrate UDP-glucose and the authors hypothesized that natural ligands modulate GALE stability in vivo [18].

### Analysis of WbgU enzymatic activity

**Fig. 3:**
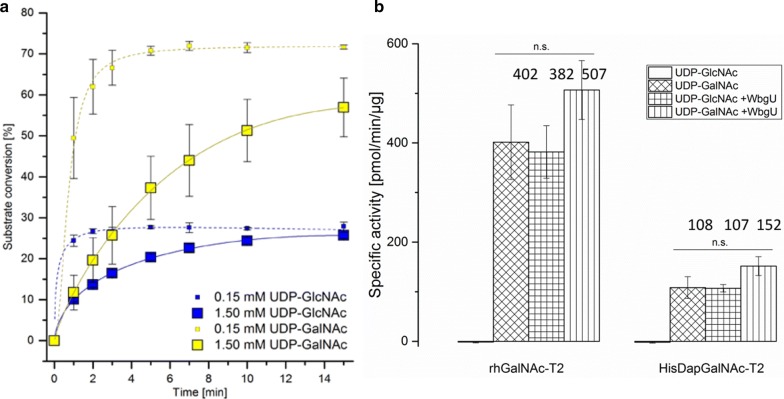
In vitro activity of WbgU analysed by capillary electrophoresis (CE) (**a**) and in combination with GalNAc-T2 using a modified glycosyltransferase assay (**b**). Substrate conversion of the sugar substrates UDP-GlcNAc (blue squares) and UDP-GalNAc (yellow squares) using 0.15 mM (small squares) and 1.50 mM (big squares) in the presence of WbgU. UDP-GlcNAc and UDP-GalNAc were separated by CE and detected at 254 nm. The mean substrate conversion was calculated based on the relative peak area of three independent experiments and results are shown including the standard deviation (**a**). The mean specific activities of recombinant human GalNAc-T2 (rhGalNAc-T2) commercially produced in NS0 cells and His-tagged GalNAc-T2 (HisDapGalNAc-T2) expressed in *E. coli* were determined in four independent experiments as duplicates using 0.5 mM UDP-GalNAc and UDP-GlcNAc, as activated sugar donors, 0.25 mM EA2 peptide acceptor substrate and 1 µg WbgU. Samples containing WbgU were incubated for 20 min at 37 °C prior to glycosyltransferase addition and subsequently incubated for 20 min at 37 °C. Results are displayed including the standard error; inserted numbers indicate the specific activities in pmol*min^−1^*µg^−1^ (**b**). *n.s.* not significant

The equilibrium reaction between UDP-GalNAc and UDP-GlcNAc in the presence of WbgU [17] was assessed by monitoring the conversion of the substrates at concentrations of 0.15 mM or 1.5 mM. After heat inactivation of WbgU, UDP-GalNAc and UDP-GlcNAc were separated by capillary electrophoresis and detected at 254 nm. When 0.15 mM UDP-sugar substrates were used, the results showed a ratio of 30:70 of UDP-GalNAc:UDP-GlcNAc (Fig. 3a). In samples containing 1.5 mM UDP-GlcNAc, a substrate conversion of about 30% was detected, whereas 57% of 1.5 mM UDP-GalNAc was converted during the reaction time, indicating a faster conversion of UDP-GalNAc (Fig. 3a). These results indicate the successful expression of functional WbgU of *P. shigelloides* in *E. coli* SHuffle^®^T7 Express. These results also correlate with data previously published for the His-tagged 4-epimerase WbgU recombinantly expressed in *E. coli* BL21(DE3) [17]. In the cited paper *K*_m_ values of 0.13 mM are reported for both substrates. At substrate saturation (1.5 mM), reaction velocities are different with the respective starting substrate [17]. The authors have described a slower conversion rate of UDP-GlcNAc than that of UDP-GalNAc based on time course experiments using multiple enzyme dilutions [17].

The in vitro activity of the epimerase combined with the commercially available glycosyltransferase rhGalNAc-T2 and purified recombinant HisDapGalNAc-T2 from *E. coli*, was assessed using a modified glycosyltransferase assay [6]. The assay is based on the detection of inorganic phosphate, which is released during the sugar transfer reaction and subsequent hydrolysis of UDP catalyzed by a phosphatase. The released phosphate is optically quantified using malachite green reagents [19]. In order to combine the epimerization reaction and the glycosyltransferase assay, a pre-incubation step of the glycosyltransferase reaction mix containing the sugar substrate and the acceptor peptide with WbgU was added. The pre-incubation step was optimised assessing different parameters, such as incubation time and enzyme concentration, to minimize a potential limitation of the glycosylation reaction by WbgU. Both glycosyltransferases were assessed in combination with either 0.5 mM UDP-GalNAc or UDP-GlcNAc as substrates, and the determined specific activities are shown in Fig. 3b. Adding WbgU and UDP-GlcNAc instead of UDP-GalNAc, the in vitro activity of WbgU was demonstrated based on the release of phosphate in the glycosylation reaction, which indicates the conversion of UDP-GlcNAc to UDP-GalNAc. Glycosyltransferase combined with WbgU and UDP-GalNAc was included as control. In the presence of UDP-GalNAc sugar substrate, the addition of WbgU increased the specific activity of rhGalNAc-T2 and HisDapGalNAc-T2 by about 26 and 40%, respectively. No activity was detected for both glycosyltransferases in the presence of UDP-GlcNAc without WbgU. Specific activities with UDP-GalNAc as a sugar substrate were roughly comparable to the values detected for the combination of WbgU and UDP-GlcNAc. In general, significantly higher activities were detected for rhGalNAc-T2 as compared to HisDapGalNAc-T2 (Fig. 3b), thus confirming previously published results [6].

### Co-expression of GalNAc-T2, WbgU, and T7Muc10

**Fig. 4:**
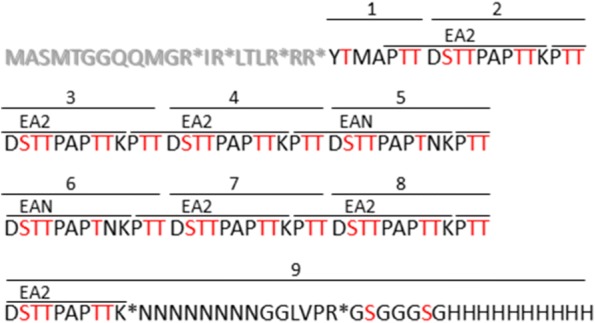
Primary structure of T7Muc10. *In silico* proteolytic (Asp-N) digestion of truncated T7Muc10 results in nine peptides (1–9) with four different sequences. The repetitive amino acid sequences EA2 and EAN are highlighted. The N-terminal peptide sequence comprising amino acids 1 to 20 could not be covered by HPLC-ESI-LTQ-OT-analysis (shown in bold and grey). Potential glycosylation sites (Ser/Thr) are highlighted in red. Predicted trypsin cleavage sites are indicated with an asterisk

**Fig. 5:**
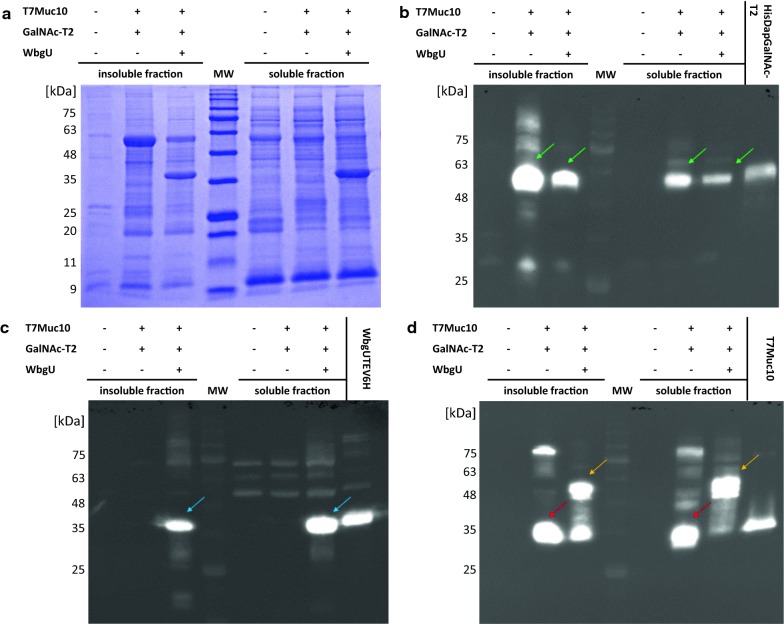
Co-expression of GalNAc-T2, WbgU, and T7Muc10. *E. coli* SHuffle^®^ T7 Express carrying plasmid pMJS9 in combination with either construct pET23d_*galNT2*_*T7Muc10* (+ GalNAc-T2, + T7Muc10, − WbgU), pET23d_*galNT2*_*T7Muc10*_*wbgU* (+ GalNAc-T2, + T7Muc10, + WbgU) or the vector control pET23d(+) (− GalNAc-T2, − T7Muc10, − WbgU) were cultured in 2 mL EnPresso^®^ B at 30 °C. Cells were harvested 24 h after IPTG induction and lysed. Insoluble and soluble fractions were separated and analysed using Coomassie-stained SDS-Gel (**a**) and Immunoblot analysis with Anti-GalNAc-T2 (**b**), Anti-WbgU rabbit serum (**c**) and Anti-His.H8 antibodies (**d**). Purified GalNAc-T2 (HisDapGalNT2, 60 kDa) (**b**), WbgU (40 kDa) (**c**), and T7Muc10 (16.5 kDa) (**d**) were included as controls. Molecular mass markers (MW) are in kDa. The presence (+) or absence (−) of *galNT2*, *T7Muc10*, and *wbgU* in the respective expression sample is indicated. Protein bands representing GalNAc-T2 are highlighted by green, WbgU by blue, and T7Muc10 by red and orange arrows

GalNAc-T2, WbgU, and protein target T7Muc10 were expressed simultaneously in a previously established *E. coli* expression system pre-/co- expressing the chaperones Erv1p and PDI, in order to probe the in vivo *O*-glycosylation activity of GalNAc-T2. The design of the target protein T7Muc10 was based on the amino acid sequence 127 to 230 of rat apomucin, resulting in a protein that comprises six repeats of the EA2 sequence (PTTDSTTPAPTTK) and two repeats of the EAN sequence (PTTDSTTPAPTNK) followed by an asparagine—glycine linker, a thrombin cleavage site, and a 10× polyhistidine-tag (Fig. 4). As a result of the employed cloning strategy, the T7 leader sequence derived from the pET23d DNA sequence was translationally fused to the N-terminus leading to the expression of T7Muc10 (Fig. 4). Expression experiments were performed including *E. coli* SHuffle^®^T7 Express pMJS9 strains harbouring either the pET23d vector without insert, a vector construct encoding GalNAc-T2 and T7Muc10 or an expression plasmid carrying the genetic information for the glycosyltransferase, the 4-epimerase and the target protein. Insoluble and soluble fractions derived from lysed cells were analysed by SDS-PAGE (Fig. 5a) and immunoblot analysis (Fig. 5b–d). The expression of GalNAc-T2 (60 kDa) and WbgU (40 kDa) was detected in both insoluble and soluble fractions (GalNAc-T2; Fig. 5b green arrows; WbgU, Fig. 5c blue arrows). Based on densitometric analysis of the Western Blot the amount of soluble WbgU was estimated at around 65% (Fig. 5c lane 7) in comparison to the corresponding fraction of 35% in the insoluble fraction (Fig. 5c lane 3). However, expressed GalNAc-T2 in the soluble fractions was less prominent (about 33% as compared to 67% in the insoluble fractions for both *E. coli* strains) (Fig. 5b). The detected molecular weight of 32 kDa for T7Muc10 was higher than the expected 16.5 kDa, indicating a potential dimerization of the protein (Fig. 5d red arrows). Interestingly, the apparent molecular mass of T7Muc10 increased significantly in the presence of WbgU (Fig. 5d orange arrows) suggesting a modification by glycosylation.

### Analysis of in vivo glycosylation of T7Muc10

**Fig. 6:**
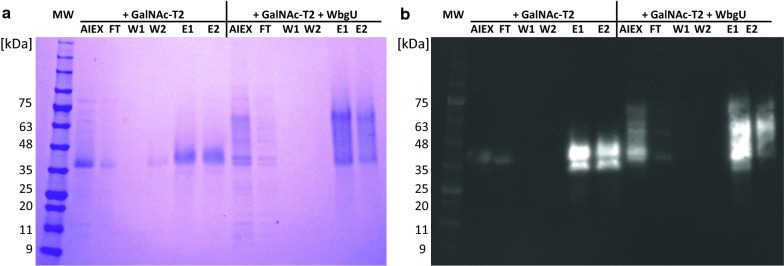
Purification of T7Muc10 using Ni–NTA affinity chromatography. Cell pellets of *E. coli* SHuffle^®^ T7 Express carrying plasmid pMJS9 in combination with vector construct pET23d_*galNT2*_*T7Muc10* or pET23d_*galNT2*_*T7Muc10*_*wbgU* expressing GalNAc-T2 and T7Muc10 or GalNAc-T2, T7Muc10 and WbgU were thawed and lysed. The soluble fractions were pre-purified using anion exchange chromatography (AIEX). Fractions of the flow-through containing T7Muc10 (AIEX) were loaded onto a Ni–NTA affinity chromatography spin column. Fractions of the flow-through (FT), the first washing step with equilibration buffer (W1), and the second washing step with washing buffer (W2) were collected. T7Muc10 was eluted by applying elution buffer twice (E1, E2). Aliquots of the fractions were analysed by SDS-PAGE stained with Coomassie (**a**) and Immunoblot treated with Anti-His.H8 antibodies (**b**). Molecular mass markers (MW) are in kDa

First attempts to capture T7Muc10 using Ni–NTA affinity chromatography failed due to unspecific binding of host cell proteins and weak interaction between protein and column (data not shown). However, the estimated high isoelectric point of the protein (pI = 10.1) allowed for an indirect anion exchange chromatography step (AIEX), thus avoiding the addition of salt and an additional dialysis step to separate the majority of host cell proteins from T7Muc10. Fractions containing pre-purified mucin derivatives were subjected to affinity chromatography and the concentrated eluates contained T7Muc10 with sufficient purity for further analysis (Fig. 6b, lanes E1 and E2). The diffuse, slightly smeared protein signal between 33 and 60 kDa detected by Western Blot analysis (Fig. 6, lanes E1 and E2) indicates the presence of putative glycosylated T7Muc10.

To verify the presence of T7Muc10 and determine potential glycosylations in the samples, Coomassie-stained protein bands containing either unglycosylated (33 kDa) or the putative glycosylated T7Muc10 (33 kDa to 60 kDa) were cut from the SDS–polyacrylamide gel. These were submitted for analysis by liquid chromatography electrospray ionization tandem mass spectrometry (LC–ESI–MS/MS). The solubilized protein was treated with trypsin prior to analysis. The detected peptides were assigned and the results indicate the presence of GalNAc-modified threonines in T7Muc10 (Table 1). Evaluation of the tandem MS data provided information about the glycosylation sites targeted by the glycosyltransferase and modified threonines (displayed as lower-case letters in bold type). Positions 3, 7, 11 and 12 of the repetitive EA2 subsequence PT**t**DST**t**PAP**tt**K in T7Muc10 were identified as possible glycosylation sites (Table 1).

**Table 1:** Detected glycosylated and fragmented peptides of T7Muc10 using ESI–MS/MS

Fragment sequence	Peptide identification probability [%]	Observed *m/z*	Actual peptide mass [Da]	Position of the modification within the EA2 peptide
PTTDSTTPAPT **t** K	97	760.87	1519.73	T12
PTTDST **t** PAPTTK	97	760.87	1519.73	T7
STTPAPT**t**KPT	100	652.83	1303.65	T12
STTPAPTTKPT**t**D	100	760.87	1519.73	T3
STTPAP**t**TKPTTD	100	760.87	1519.72	T11

T7Muc10 contains six repeating EA2 peptide sequences (PTTDSTTPAPTTK). To highlight adjacent EA2 repetitions within the detected fragments, amino acids of the neighbouring EA2 amino acid sequence are underlined. Identified modified threonines carrying GalNAc residues are displayed as lower-case letters in bold type

**Fig. 7:**
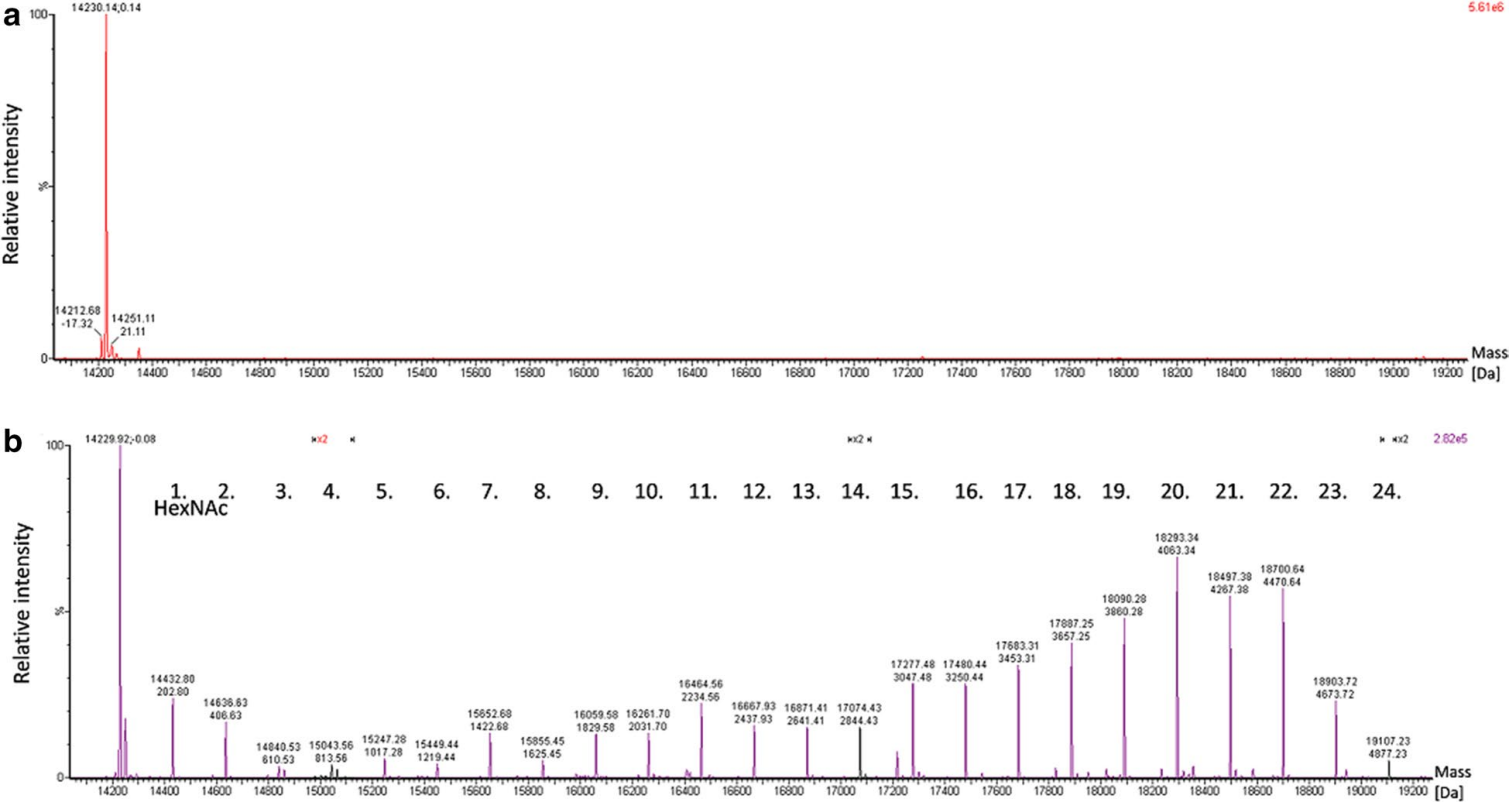
UPLC-ESI-QTOF-MS analysis of intact unglycosylated and glycosylated T7Muc10. Purified protein samples were dialysed, filtered, and analysed by UPLC-ESI-QTOF mass spectrometry. Charge deconvolution of MS spectra was realized using the MaxEnt 1 algorithm. Charge-deconvoluted MS spectra of intact N-terminally truncated T7Muc10 (AS 21–159) (**a**) and glycosylated T7Muc10 (**b**) are shown. The average molecular mass (Da) of respective proteoforms is given. Each of the up to 24 *O*-linked β-*N*-acetylgalactosamines results in a mass increment of 203.2 Da

To examine the protein expression and overall glycosylation of T7Muc10, samples containing unglycosylated and potentially glycosylated T7Muc10 were subjected to intact mass determination by ultra-performance liquid chromatography coupled with electrospray ionization—quadrupole time-of-flight mass spectrometry (UPLC-ESI-QTOF-MS). The intact mass analysis revealed an average molecular mass of T7Muc10 of 14,230 Da, instead of the expected 16,531 Da calculated from its theoretical amino acid sequence (Fig. 7a). This result corresponds to a truncated version of T7Muc10 (AS 21 to 159), lacking the first 20 N-terminal amino acids measured with a mass deviation of 68 ppm. The presence of truncated recombinantly expressed T7Muc10 has been confirmed by determining amino acids 1 to 5 using N-terminal Edman sequencing. Whether the absence of the N-terminal region was due to cleavage by proteases in the *E. coli* cytoplasm during expression or a result of proteolytic degradation during purification procedures is still unclear. LC–MS analysis of potentially glycosylated T7Muc revealed different glycosylation stages, ranging from zero up to 24 attached sugar moieties (Fig. 7b). Each attached moiety adds 203.2 Da in mass, which is consistent with the presence of GalNAc. Up to 24 of the 57 potential *O*-glycosylation sites of the N-terminally truncated T7Muc10 (AS 21–159) were glycosylated, increasing the average molecular mass by roughly 4877 Da.

To assess the glycosylation level of EA2- and EAN repetitions, unglycosylated and potentially glycosylated T7Muc10 samples were treated with endoproteinase Asp-N (Fig. 4). These samples were analysed by HPLC-ESI linear trap quadrupole-orbitrap (LTQ-OT)-MS and MS/MS. Different glycosylation levels of EA2- and EAN-peptides ranging from zero to four attached GalNAc moieties were detected in the MS data (Table 2). Some peptides with the same molecular mass and consequently equal glycosylation level were detected at different retention times indicating different localizations of the attached GalNAc moieties (Table 2).

**Table 2:** HPLC-ESI-LTQ-OT-MS/MS analysis of glycosylated T7Muc10

Peptide description	Peptide sequence	Retention time [min]	*m/z* measured	[M + H]^+^_theor_	Charge state	Δm [ppm]	GalNAc moieties	Relative abundance [%]
1	YTMAPTT	16.93	784.355	784.355	+ 1	− 0.98	0	88.21
		18.54	987.434	987.434	+ 1	0.61	1	11.80
2/3/4/7/8	DSTTPAPTTKPTT	11.65	1317.653	1317.653	+ 1	0.62	0	27.64
		10.68	1520.733	1520.733	+ 1	− 0.40	1	40.35
		9.14	1723.812	1723.812	+ 1	0.31	2	25.10
		10.83	1723.812	1723.812	+ 1	− 0.32	2	4.34
		10.47	1926.891	1926.891	+ 1	− 0.18	3	2.22
		10.03	1065.489	2129.971	+ 2	− 0.37	4	0.36
5/6	DSTTPAPTNKPTT	10.42	1330.649	1330.649	+ 1	0.23	0	21.00
		9.82	1533.728	1533.728	+ 1	0.39	1	36.34
		8.38	1736.807	1736.807	+ 1	0.13	2	27.71
		9.98	1736.807	1736.807	+ 1	− 0.07	2	12.00
		8.00	1939.887	1939.887	+ 1	− 0.01	3	2.45
		9.93	1939.887	1939.887	+ 1	− 0.96	3	0.43
		9.47	1071.987	2142.966	+ 2	− 0.14	4	0.07
9	DSTTPAPTTKNNNNNNNNGGLVPRGSGGGSGHHHHHHHHHH	n.d.		4339.9583		–	0	0

Proteolytic (AspN) peptides of glycosylated T7MuC10 were identified via HPLC-ESI-LTQ-OT-MS/MS analysis. The theoretical monoisotopic mass increases by 203.07937 u per attached *O*-acetylgalactosamine (C8O5NH13) moiety. Relative quantification of peptides was conducted using the MassMap^®^ software. Extracted ion chromatograms (XIC) of MS signals were generated using a mass tolerance of 5 ppm. Relative peptide abundances of glycosylated and non-glycosylated peptides were calculated from the respective peptide peak areas. The C-terminal peptide was not detected (n.d.)

Evaluation of the MS/MS spectra revealed monosaccharide modifications at positions 2, 3, 6, 7, 11 and 12 of the tandem EA2. Monosaccharide modifications were also found at positions 2, 6, 7 and 11 of the EAN sequence. Interestingly, a single modified serine residue was also identified. This was detected at position 5 of the tandem EA2 sequence, adjacent to the asparagine- glycine linker (Additional file 1: Table S1).

## Discussion

To establish an *E. coli* strain as cell factory for the production of recombinant glycoproteins, the functionality of the recombinant human GalNAc-T2 (shown in a previously described expression system) has been demonstrated in vitro [6]. In the present work, this expression strain was further modified to ensure the biosynthesis of the sugar substrate in the host background. The absence of the necessary activated sugar substrate (UDP-GalNAc) in laboratory *E. coli* strains requires the co-expression of an additional UDP-GlcNAc 4-epimerase to generate UDP-GalNAc from UDP-GlcNAc [9, 20]. Therefore, we introduced the 4-epimerase WbgU into the previously described *E. coli* SHuffle^®^T7 Express strain. The 4-epimerase was successfully expressed as judged by immunoblot analysis using polyhistidine-tag targeting antibodies. The protein was subsequently isolated and analysed. The in vitro activity of WbgU in combination with GalNAc-T2 was demonstrated using a modified glycosyltransferase assay. As expected from previously published results [21], UDP-GlcNAc is not a substrate for GalNAc-T2. Measured activities tended to be higher when WbgU and GalNAc-T2 were used in combination with UDP-GalNAc. However, the comparison of the activities determined in this assay, whether in the presence of WbgU or not, is rather difficult to determine—due to the ongoing epimerization reaction. By adding the UDP-GlcNAc 4-epimerase to the assay, the ratio of UDP-GlcNAc:UDP-GalNAc during the glycosylation reaction is unknown. In general, samples with both WbgU and UDP-GlcNAc showed comparable activities to samples with UDP-GalNAc, confirming the functionality of the 4-epimerase and the glycosyltransferases in the described assay in vitro.

The usefulness of *E. coli* SHuffle^®^T7 Express for the expression of complex proteins containing disulfide bonds has already been demonstrated [6, 22, 23]. In vivo glycosylation has also been shown [9]. In the expression system published previously, an N-terminally truncated version of GalNAc T2, inserted under the control of the T7 promoter, has been expressed in combination with the 4-epimerase derived from *P. aeruginosa* including its endogenous promoter in *E. coli* SHuffle^®^T7 Express without IPTG induction [9]. Glycosylation of a mucin fusion protein has been assessed by lectin in Western blot assays. The analysis of an anti-TNF-α antibody fragment fusion to the 15 amino acid mucin-derived small peptide CDNKPAPGSTAPPAA by LC/MS revealed an increase in molecular weight of 203 Da, consistent with the presence of a single GalNAc moiety [9]. The present work describes a different approach to isolate a robust expression strain, aiming towards high-level in vivo glycosylation of complex proteins. The successful co-expression of GalNAc-T2 and WbgU—under the control of the T7 promotor in the presence of the two chaperones Erv1p/PDI in *E. coli* SHuffle^®^T7 Express with IPTG and l-arabinose induction—was demonstrated by immunoblot analysis with Anti-GalNT2 antibodies and polyclonal Anti-WbgU serum. The potential of the *E. coli* strain was assessed by co-expressing the target protein T7Muc10, containing 60 putative glycosylation target sites, with 49 threonine- and 11 serine residues. In mammals, mucins form homooligomers via inter-molecular disulfide bonds between cysteine-rich domains at both ends of the proteins [24]. Using porcine submaxillary mucin (PSM) as an example, *N*-glycosylation is followed by the dimerization of PSM via disulfide bond formation between the C-terminal domains [25–27]. Subsequently, *O*-glycosylation and N-terminal multimerization of the dimers [25–27] occur, mediated by disulfide bonds between the N-termini [27]. Furthermore, non-covalent dimerization—probably by means of hydrophobic interactions—of domains of the colonic human MUC2 mucin containing cysteines has been identified [28]. For MUC5AC and MUC5B, higher molecular weight aggregates have been detected using SDS-PAGE, under both reducing and non-reducing conditions [29]. The synthetic T7Muc10 target protein used in the present work does not contain cysteine residues. As a result, the observed putative dimerization of the protein, indicated by the 33 kDa protein band in SDS-PAGE analysis, may be due to different interactions.

The potential glycosylation of T7Muc10 was subsequently verified by ESI–MS/MS. The results showed the presence of different glycosylation patterns with varying target sites and different amounts of monosaccharide moieties. These results are consistent with the presence of GalNAc residues attached to the glycosylated mucin. No molecular shift of T7Muc10 was detected when GalNAc-T2 and WbgU were not co-expressed. Putative glycosylation sites were postulated for di- or triglycosylated EA2 and EAN sequences based on MS1 measurements. These sites were confirmed by subsequent MS2 analysis. Although UDP-galactose has been postulated as an alternative sugar substrate for GalNAc-T2 [21, 30], no sugar moieties attached to T7Muc10, which correlate with galactose residues were detected. GalNAc-T2 has been classified as an early transferase, preferring mono- or un-glycosylated protein substrates [14]. Previous in vitro studies with GalNAc-T2 have identified Thr7 in the synthetic EA2 peptide sequence [14, 15] and Thr9 in the MUC5AC peptide as preferred glycosylation sites [31]. Both threonines are located in the central areas of the peptide sequences. We have recently demonstrated the presence of up to three GalNAc residues attached by GalNAc-T2 to the EA2 peptide in vitro [6]. Differences in the respective results reflect the use of a short EA2 peptide in a chemically defined in vitro environment, as compared to the glycosylation of the larger artificial T7Muc10 derivative in a complex cytoplasmic environment in vivo. The data presented in this work indicates the truncated T7Muc10 glycosylation at different positions of the 57 possible target sites, in various degrees and combinations, leading to a multitude of different glycopeptides; thus demonstrating a high level mucin-type *O*-glycosylation. Furthermore, the results emphasize the potential of the presented glycosylation platform for in vivo glycosylation of commercially relevant biopharmaceuticals.

## Conclusion

High level in vivo *O*-glycosylation of a MUC10 derived protein was demonstrated using the *E. coli*-based expression platform presented in this work. *O*-glycosylation sites were modified in different combinations, and to various degrees, by the co-expression of the target protein, the human glycosyltransferase GalNAc-T2, the 4-epimerase WbgU, and the redox folding helper proteins PDI and Erv1p in *E. coli* SHuffle^®^ T7 Express. The functionality of GalNAc-T2 and WbgU was also demonstrated in vitro, and the soluble expression of both enzymes was confirmed. Threonines at position 2, 3, 6, 7, 11 and 12 of the tandem EA2, correlating with threonines at position 2, 6, 7 and 11 of the EAN sequence, and a single serine residue at position 100 in the truncated T7Muc10, were identified as potential glycosylation sites in vivo. A maximum of 4 glycosylated sites in a peptide EA2/EAN repeat were detected. The value of the presented robust glycosylation platform will be further verified by introducing pharmaceutically relevant target proteins to assess in vivo glycosylation of important biopharmaceuticals. The approach might further enable the production of glycosylated protein based drugs that are easily accessible for site-specific in vitro polyethylene glycol modification (PEGylation), which would allow for the generation of long-lasting dosage forms in the future [9, 32].

## Materials and methods

### Bacterial strains

The *Escherichia coli* strains NovaBlue (Novagen, Merck Millipore, Germany) and SHuffle^®^T7 Express (C3028H, New England Biolabs, Germany) were routinely grown as described previously [6]. Transformation and selection were carried out following protocols already published [6]. Experiments to produce WbgU were carried out using TB Medium supplemented with 0.5% d-glucose (Glc). For the production of unglycosylated T7Muc10 5010 Medium (50 g/L yeast extract, 10 g/L peptone, 0.492 g/L magnesium sulfate heptahydrate) was used. Glycosylated T7Muc10 was expressed as described previously in a 24-deepwell plate containing 2 mL EnPresso^®^ B medium in each well and pooled for purification [6].

### Plasmids

Plasmid DNA of pET-23a(+) and pET-23d(+) (both obtained from Merck Millipore (Germany)) was prepared using the Roti^®^-Prep Plasmid MINI kit (Carl Roth GmbH & Co. KG, Germany), DNA fragments were isolated from agarose gel blocks employing the Monarch Gel Extraction Kit (New England Biolabs, Germany) and PCR products were purified using the PCR Clean Up Extraction Kit (GeneOn, Germany). Primers were obtained from Thermo Scientific, Germany. Synthesised DNA fragments of *wbgU* and *muc10* were purchased from GeneArt^®^ Gene Synthesis and supplied as shuffle vector pMA-T-derived constructs (Life Technologies GmbH, Germany). Plasmid pMJS9 [8] was kindly provided by Prof. Dr. L. W. Ruddock.

The DNA sequence encoding the UDP-GlcNAc 4-epimerase WbgU derived from *Plesiomonas shigelloides* [WbgU, GenBank:AAG17409.1, UniProtID:Q7BJX9] was adapted for expression in an *Escherichia coli* strain background [33]. The restriction sites *Xba*I and *Xho*I were added at the 5′ and 3′ end of the DNA fragment, respectively. Gene fragments were amplified by PCR using Primer 1 (CTC TCT AGA AAT AAT TTT), Primer 2 (ATT CTC GAG TGA ACC TTT CAG AAA ACG AAC), dNTP’s (GeneOn, Germany) and Phusion Polymerase (Thermo Scientific, Germany). The stop codon TAA was replaced with the serine codon TCA to translationally fuse the His-tag sequence of the pET-23a vector. The isolated PCR product was digested with *Xba*I and *Xho*I and the resulting 1.1 kb fragment was purified and ligated with vector pET-23a treated with the same restriction enzymes. The DNA was subsequently used to transform *E. coli* NovaBlue and construct pET23a(+)_*wbgU6xhis* was isolated encoding WbgU with the sequence SLEHHHHHH* translationally fused to the C-terminal glycine. Annealed oligo cloning with the 5′-phosphorylated Primer 3 (TCG AGA ACC TGT ATT TTC AGA GCG) and 4 (TCG ACG CTC TGA AAA TAC AGG TTC) was used to introduce the specific recognition sequence of the TEV protease into the *Xho*I restriction site of pET23a(+)_*wbgU6xhis*. Consequently, the C-terminus of WbgU was extended to SLENLYFQ’SVEHHHHHH*, with the apostrophe representing the cleavage site and the asterisk the stop codon. The resulting construct pET23a(+)_*wbgUTEV6H* was verified by restriction digests and DNA sequence analysis.

Plasmid pET23a_*galNT2* was constructed by introducing *galNT2* from the donor plasmid pMK-RQ [6] into pET23a(+) using *Nde*I and *Xho*I restriction sites. Ligation of the *galNT2*-fragment treated with *Xba*I and *Spe*I restriction endonucleases and the *Xba*I linearized pET23d(+) vector resulted in isolating construct pET23d_*galNT2*. The cloning steps to assemble vector pET23d_*galNT2*_*T7Muc10*_*wbgU* were carried out by Synbio Technologies LLC, NJ, USA, including the *Sac*I/*Not*I and *Bam*HI/*Eco*RI restriction digestion of the pMA-T vectors carrying *wbgU* and *muc10*, respectively, and the ligation of the isolated DNA fragments encoding WbgU and Muc10 with the corresponding enzymes-digested vector fragment of pET23d_*galNT2*. The N-terminus of Muc10 was translationally fused to the T7-tag encoded on the pET23d_*galNT2* vector resulting in T7Muc10. Plasmid pET23d_*galNT2*_*T7Muc10* was obtained by deleting *wbgU* from pET23d_*galNT2*_*T7Muc10*_*wbgU* using *Eco*RI/*Xho*I restriction and 5′-phosphorylated Primer 5 (AAT TCC AAT TGT GAG CGG C) and 6 (TCG AGC CGC TCA CAA TTG G) to religate the vector.

The constructs were verified by restriction digests and the inserted fragments were confirmed by DNA sequence analysis.

### Expression of WbgU, HisDapGalNAc-T2, and T7Muc10

For large-scale cultivation, a bioreactor BIOSTAT^®^ C-DCU with a working volume of 10 L was used. Temperature, pH, and aeration were set to 30 °C, pH 7, and 6 slpm, respectively. The oxygen saturation was kept constant at 60% via agitation starting with a lower limit of 100 rpm. The pH was adjusted with 1 M NaOH and 1 M H_2_SO_4_. *E. coli* SHuffle^®^T7 Express harboring pET23a(+)_*wbgUTEV6H* and pMJS9 was pre-cultured in two baffled shake flasks with 750 mL TB medium at 30 °C and 140 rpm until OD_600_ > 2 was reached. Subsequently, the bioreactor was inoculated and at OD_600_ of 1 a feed was started till a total volume of 20 L. l-arabinose was added to a final concentration of 0.5%. The culture was harvested 6.5 h post inoculation and the pellet with a wet cell weight of 257 g was stored at − 20 °C.

Protein isolation of HisDapGalNAc-T2 was carried out using *E. coli* SHuffle^®^T7 Express harboring pET23d(+)_*HisDapGalNAcT2* [6] and pMJS9. The cells were pre-cultured in 5 mL LB medium supplemented with 0.5% Glc at 30 °C and 175 rpm. A baffled shaking flask with a working volume of 25% EnPresso^®^ B medium was inoculated to OD_600_ of 0.01 and reagent A was added at a ratio of 1:2000. The culture was incubated at 30 °C and 170 rpm. 17 h post-inoculation booster tablets (1 per 50 mL), l-arabinose (final concentration 0.5%) and 17.5 h post-inoculation IPTG (final concentration 1 mM) were added. Cells from a 125 mL culture were harvested 24 h post-arabinose-induction and a pellet with a wet cell weight of 2.2 g was obtained and stored at − 20 °C.

To produce large amounts of unglycosylated T7Muc10, *E. coli* SHuffle^®^T7 Express harboring pET23d(+)_*galNT2_T7Muc10* and pMJS9 was pre-cultured in a 2 L baffled shaking flask with 1 L 5010 medium at 30 °C and 160 rpm until an OD_600_ higher than 2 was reached. Two 5 L baffled shaking flasks with 900 mL 5010 medium were inoculated each with 350 mL cell culture and incubated at 30 °C and 170 rpm. When the OD_600_ was higher than 1, IPTG and l-arabinose were added with final concentrations of 1 mM and 0.5%, respectively. Cells were harvested 4 h post-induction and a pellet with a wet cell weight of 8.4 g was obtained and stored at − 20 °C.

Glycosylated T7Muc10 was produced by cultivating *E. coli* SHuffle^®^T7 Express harboring pET23d(+)_*galNT2_T7Muc10_wbgU* and pMJS9 in two 24-deepwell plates filled with 2 mL EnPresso^®^ B medium per well and incubated at 30 °C and 200 rpm. Expression experiments were carried out as described for HisDapGalNAc-T2. A pellet with a wet cell weight of 1.26 g was obtained and stored at – 20 °C.

### Protein purification

1 g of the respective expression culture cell pellet was resuspended in 2 mL extraction buffer (50 mM tris, 300 mM NaCl; pH 8 for Ni–NTA affinity chromatography or 50 mM tris pH 9 for anion exchange chromatography) containing 112 μL lysozyme (10 mg/mL) and 1 μL DNAseI (10 kU/mL). The bacterial suspension was kept on ice for 2 h and sonicated for 2.5 min cooled on ice. The cell lysate was centrifuged at 4 °C, 18,000×*g* for 10 min, the supernatant collected, pH-value confirmed, and the liquid was subsequently passed through a 0.45 μM filter (Titan3™ PVDF, Life Technologies GmbH, Germany).

The polyhistidine-tagged proteins HisDapGalNAc-T2 and WbgUTEV6H were purified on a HisTrap HP 1 mL column with an ÄKTA Purifier system (GE Healthcare Life Sciences). The column was equilibrated with buffer A1 (50 mM tris, 300 mM NaCl; pH 8) and the sample was loaded. For HisDapGalNAc-T2 the column was washed with 22% buffer B1 (50 mM tris, 300 mM NaCl, 500 mM imidazole; pH 8) and the protein eluted with 55% buffer B1. Fractions containing HisDapGalNAc-T2 were dialysed (Slide-A-Lyzer™, Thermo Scientific, Rockford, IL) in 25 mM tris, 150 mM NaCl; pH 7.3 and stored at 4 °C. For WbgUTEV6H the column was washed with 10% and the protein eluted with 16% buffer B1. Fractions were collected during the elution procedure and samples containing WbgUTEV6H were pooled and dialysed against 50 mM tris, 300 mM NaCl; pH 8.0. To remove the polyhistidine tag, 2 µl TEV protease (Protean s.r.o., Dobra Voda, Czech Republic) per 100 µg protein was added and subsequently incubated at 30 °C for 3 h. Using Ni–NTA affinity chromatography, TEV protease-treated WbgU was obtained in the flow-through. Fractions containing WbgU were concentrated using Vivaspin™ columns (MWCO 10 kDa, GE Healthcare Life Sciences) and dialysed against 50 mM Tris; pH 8.0. For long-term storage at 4 °C, 2× storage buffer (50 mM tris, 1 M trimethylamine *N*-oxide, 1 M trehalose; pH 8.0) was added in a 1:2 ratio.

T7Muc10 was purified using anion exchange chromatography (AIEX). Four 1 mL CaptoAdhere columns (GE Healthcare Life Sciences) in serial arrangement were equilibrated with buffer A2 (50 mM tris; pH 9) and the sample was loaded. The protein was recovered in the fractions of the flow-through. The samples were pooled, titrated to pH 8.0, and concentrated using Ni–NTA affinity spin columns (Qiagen, Hilden, Germany), according to the manufacturer’s instructions employing buffer A1 and B1. Concentrated fractions were pooled, dialysed against 20 mM tris; pH 8.0, and filtered (0.2 µm, Phenomenex, Aschaffenburg, Germany).

### Analysis of purified protein

SDS-PAGE and immunoblot analyses were carried out using standard protocols with dithiothreitol (DTT) as the reducing agent. Densitometric analysis was performed employing Fusion-FX software (Vilber Lourmat, Germany). For Western blot analysis, proteins were transferred to polyvinylidene fluoride membranes (PVDF, Bio-Rad Laboratories Inc., USA) and treated with mouse Anti-human GALNT2 antibodies (H00002590-A01, Acris Antibody GmbH, Germany), 6x-His tag monoclonal antibody HIS.H8 (Thermo Scientific, Rockford, IL) or Anti-WbgU serum (WbgU-immunized rabbits, Pineda antibody service, Berlin, Germany). Binding was detected with horseradish peroxidase (HRP)-labeled secondary antibodies (Jackson ImmunoResearch, West Grove, PA) and chemiluminescence was assessed using Clarity™ or WesternBright ECL solutions.

Purified WbgU was analysed by circular dichroism (CD) spectroscopy to determine folding state and thermal transitions of the protein in the presence and absence of the activated sugar substrates UDP-GlcNAc and UDP-GalNAc (25 mM each). Spectra of the filtered solution (NanoSep^®^ MF 0.2 mM, Pall) containing 0.6 mg/mL WbgU in 20 mM sodium phosphate buffer (pH 7.75) were recorded in a 715 CD-spectropolarimeter (Jasco, Hachioji, Japan) at 25 °C. Spectra were measured from 190–260 nm with wavelength steps of 0.1 nm and a scan speed of 50 nm per minute. The averaged signal from four scans was corrected for the buffer signal. Thermal transitions were recorded at 220 nm with a step size of 0.1 °C and a thermal slope of 1 °C per minute. The data was fitted using a Boltzmann equation $y = \frac{{{\text{A}}_{1} - {\text{A}}_{2} }}{{1 + e\left( {x - x_{0} } \right)/dx}} + {\text{A}}_{2}$ to obtain the transition point (OriginPro 8, OriginLab, Northampton, Massachusetts, USA).

For N-terminal Edman sequencing of WbgU and T7Muc10, solutions containing about 80 µg of protein in 20 mM sodium phosphate pH 7.75 were supplied for sequencing (Proteome Factory, Berlin, Germany).

### Protein activity assays

The substrate conversion of the sugar substrates UDP-GlcNAc and UDP-GalNAc in the presence of WbgU was analysed by capillary electrophoresis (CE). 0.15 mM and 1.5 mM UDP-HexNAc (*N*-Acetylhexosamine) concentrations in 20 mM tris; pH 8.5 were incubated at 37 °C. 80 µl samples were taken at 8 different time points (0, 1, 2, 3, 5, 7, 10 and 15 min) and inactivated at 65 °C for 5 min. After the first sampling, 3.5 µg WbgU was added to the remaining 560 µL reaction volume and subsequently, samples were taken at the next 7 time points. Heat inactivated samples were stored at 4 °C. Prior to analysis, samples were centrifuged. UDP-GlcNAc and UDP-GalNAc were separated employing the CE-UV 7100 system (Agilent Technologies, Santa Clara, CA), equipped with a fused silica capillary (50 µm inside diameter, 65 cm total length and 56.5 cm effective separation length), and using a 20 mM sodium borate, 64 mM boric acid; pH 9.0 buffer system at 30 kV. Samples were injected at 30 mbar for 10 s and detected at 254 nm. Monitored data was evaluated with openLAB CDS CehmStation C.01.07.

The activity of WbgU in combination with HisDapGalNAc-T2 and rhGalNAc-T2 (7507-GT-020, R&D Systems Europe Ltd., UK) was monitored using a modified version of the previously described glycosyltransferase activity assay (EA001, R&D Systems Europe Ltd., UK) [6] by replacing UDP-GalNAc with UDP-GlcNAc and WbgU (1 µg). Solutions containing WbgU were incubated for 20 min at 37 °C prior to the addition of glycosyltransferases.

### Mass spectrometry analysis

After separation by SDS-PAGE and visualization of the proteins by Coomassie staining, protein bands containing T7Muc10 and potentially glycosylated T7Muc10 were cut out and submitted to the life science center of the University of Hohenheim (Germany) for protein identification via LC–ESI–MS/MS analysis. Excised gel pieces were *in*-*gel* digested with trypsin prior to LC–ESI–MS/MS analysis.

Alternatively, purified T7Muc10 and potentially glycosylated T7Muc10 were subjected to *in*-*solution* digestion. Sequencing grade Asp-N (Promega Corp., Madison, WI) was added to a final enzyme:substrate ratio of 1:100 (w/w) and the samples were incubated for 18 h at 37 °C. The resulting peptide mixtures were analysed by LC–MS on a Shimadzu Prominence HPLC system (Shimadzu, Kyoto, Japan) coupled to an LTQ-Orbitrap XL mass spectrometer (Thermo Fisher Scientific) equipped with a heated electrospray ionization source (Thermo Fisher Scientific). Chromatographic separation was carried out on a Symmetry C18 column (2.1 mm × 150 mm, 3.5 µm, 100 Å, Waters Corp., Milford, MA). Peptide elution was performed by applying a mixture of solvents A and B. Solvent A consisted of 0.1% (v/v) formic acid and solvent B was 0.1% (v/v) formic acid in 85% (v/v) acetonitrile (MS grade, Honeywell). Separations were performed by applying a linear gradient of 2% to 30% solvent B over 45 min. The column was kept at 40 °C and the flow rate was set to 250 µl/min. MS data were acquired throughout the duration of the gradient using the data-dependent MS/MS mode. Each high-resolution full scan (*m/z* of 200–2000 and resolution of 60,000) in the Orbitrap analyzer was followed by five product ion scans (collision-induced dissociation-MS/MS) in the linear ion trap for the five most intense signals of the full scan mass spectrum (isolation window, 2.0 Th). Dynamic exclusion (repeat count: 1, repeat duration: 30 s; exclusion duration: 30 s) was enabled to allow MS/MS-analysis of less abundant precursor ions. Peptide identification was performed using the Proteome Discoverer 1.4 (Thermo Fisher Scientific). MS and MS/MS data of precursor ions in the *m/z* range 500–5000 were searched against an *in*-*house* database using SEQUEST. Mass accuracy was set to 5 ppm and 0.8 Da for precursor and fragment ions, respectively. Label-free relative quantification of peptides was conducted using the MassMap ^®^ software (MassMap GmbH & Co. KG, Wolfratshausen, Germany). For all peptides of interest, extracted ion chromatograms (XIC) of MS signals were generated using a mass tolerance of 5 ppm. Relative peptide abundances of glycosylated and non-glycosylated peptides were calculated from the respective peptide peak areas.

Intact mass determination of T7Muc10 and potentially glycosylated T7Muc10 in 20 mM tris pH 8.0 was performed on an ACQUITY UPLC System (Waters Corp., Milford, MA) coupled to a Xevo G2 QTof mass spectrometer (Waters Corp., Milford, MA) equipped with an ESI source (ZSpray, Waters Corp., Milford, MA). Reverse-phase desalting and protein separation was carried out on a BioSuite™ Phenyl column (1000 Å, 10 µm, 2.0 mm × 75 mm, Waters Corp., Milford, MA). The mobile phase A was 0.1% (v/v) formic acid in water, while mobile phase B contained 0.1% (v/v) formic acid in acetonitrile. The flow rate and column temperature were maintained at 0.150 mL/min and 65 °C, respectively, throughout the run. Chromatographic separation was performed by applying a linear gradient from 10 to 35% solvent B over 15 min. Mass spectrometric analysis was carried out in positive ion mode. The desolvation gas and source temperatures were set to 240 °C and 115 °C, respectively. The capillary and cone voltages were set at 3000 and 35 V, respectively. The instrument was calibrated in the *m/z* range of 400–4000 using NaI. The deconvolution of ESI mass spectra of intact protein was performed by MassLynx V4.1 (Waters Corp., Milford, MA) using the MaxEnt 1 algorithm. For deconvolution, the input mass range from *m/z* 500–2000 was used with the following MaxEnt 1 parameters: output mass range from 5000 to 55,000 Da; minimum intensity ratio left and right, 50%; width at half height for uniform Gaussian model, 0.6; number of iterations, 20.

## Additional file

**Additional file 1.** HPLC-ESI-LTQ-OT-MS/MS analysis of glycosylated T7Muc10. Proteolytic (AspN) peptides of glycosylated T7MuC10 detected using HPLC-ESI-LTQ-OT-MS analysis were further fragmented. To highlight adjacent EA2 (PTTDSTTTPAPTTK) or EAN (PTTDSTTTPAPTNK) repetitions within the detected fragments, amino acids of the neighbouring EA2 or EAN amino acid sequence are underlined. Identified modified threonines and serines carrying GalNAc residues using Proteome Discoverer 1.4 are lowercased and marked bold. The posterior error probability (PEP, the lower the better) and cross correlation (Xcorr, the higher the better) were included to evaluate the quality of the generated results.
