# Supplementary material for: High level in vivo mucin-type glycosylation in Escherichia coli
Source: Microb Cell Fact. 2018 Oct 26;17:168. doi: 10.1186/s12934-018-1013-9 (PMC6202839; doi:10.1186/s12934-018-1013-9)
Supplement: Supplementary file 1 — Additional file 1. HPLC-ESI-LTQ-OT-MS/MS analysis of glycosylated T7Muc10. Proteolytic (AspN) peptides of glycosylated T7MuC10 detected using HPLC-ESI-LTQ-OT-MS analysis were further fragmented. To highlight adjacent EA2 (PTTDSTTTPAPTTK) or EAN (PTTDSTTTPAPTNK) repetitions within the detected fragments, amino acids of the neighbouring EA2 or EAN amino acid sequence are underlined. Identified modified threonines and serines carrying GalNAc residues using Proteome Discoverer 1.4 are lowercased and marked bold. The posterior error probability (PEP, the lower the better) and cross correlation (Xcorr, the higher the better) were included to evaluate the quality of the generated results. [file 12934_2018_1013_MOESM1_ESM.docx]

**Table S1. HPLC-ESI-LTQ-OT-MS/MS analysis of glycosylated T7Muc10.** Proteolytic (AspN) peptides of glycosylated T7MuC10 detected using HPLC-ESI-LTQ-OT-MS analysis were further fragmented. To highlight adjacent EA2 (PTTDSTTTPAPTTK) or EAN (PTTDSTTTPAPTNK) repetitions within the detected fragments, amino acids of the neighbouring EA2 or EAN amino acid sequence are underlined. Identified modified threonines and serines carrying GalNAc residues using Proteome Discoverer 1.4 are lowercased and marked **bold**. The posterior error probability (PEP, the lower the better) and cross correlation (Xcorr, the higher the better) were included to evaluate the quality of the generated results.

| Peptide sequence | Retention time [min] | [M+H]^+^_theor_ | Charge state | Δm [ppm] | PEP | Xcorr | Position of modification within the EA2/EAN peptide |
| --- | --- | --- | --- | --- | --- | --- | --- |
| D**s**TTPAPTTKNNN | 5.19 | 1563.713 | 2 | -0.10 | 5.39 x 10^-5^ | 1.32 | S5 |
| DS**t**TPAPTTKNNNNNNN | 5.20 | 2019.886 | 3 | 0.39 | 2.32 x 10^-9^ | 0.76 | T6 |
| DS**t**TPAPTTKN | 5.26 | 1335.628 | 2 | 0.47 | 1.38 x 10^-3^ | 1.10 | T6 |
| D**s**TTPAPTTKNN | 5.29 | 1449.670 | 2 | -0.30 | 6.21 x 10^-5^ | 1.49 | S5 |
| ST**t**PAPtNKPTT | 6.78 | 1621.781 | 2 | 0.28 | 1.38 x 10^-3^ | 1.10 | T7 |
| S**tt**PAPTTKPTT | 7.39 | 1608.784 | 3 | -0.55 | 3.75 x 10^-3^ | 0.37 | T6, T7 |
| DS**tt**PAP**t**NKPTT | 8.03 | 1939.886 | 2 | -0.20 | 1.38 x 10^-3^ | 0.59 | T6, T7, T11 |
| S**t**TPAPTNKPTT | 8.05 | 1418.700 | 3 | -0.53 | 1.38 x 10^-3^ | 0.69 | T6 |
| TTPAP**t**TKPTT | 8.22 | 1318.673 | 2 | -0.60 | 1.38 x 10^-3^ | 1.64 | T11 |
| DSTTPAPT**t**KNNNNNNNNGGLVPRGSGGGSGHHHH | 8.35 | 3720.682 | 6 | -0.53 | 2.47 x 10^-3^ | 1.16 | T12 |
| DST**t**PAP**t**NKPTT | 8.57 | 1736.808 | 2 | 0.35 | 1.38 x 10^-3^ | 1.17 | T7, T11 |
| STTPAP**t**TKPTT | 8.74 | 1405.705 | 2 | -0.25 | 2.97 x 10^-7^ | 1.22 | T11 |
| TTDST**t**PAP**tt**KP | 8.83 | 1926.892 | 2 | 0.44 | 1.38 x 10^-3^ | 0.80 | T7, T11, T12 |
| DSTTPAPTTKPT**t** | 9.10 | 1520.732 | 2 | -0.21 | 4.20 x 10^-4^ | 1.41 | T3 |
| DSTTPAP**t**NKPTT | 10.26 | 1533.728 | 2 | -0.22 | 1.38 x 10^-3^ | 1.43 | T11 |
| DST**t**PAPtTKPTT | 10.80 | 1723.812 | 2 | 0.22 | 1.19 x 10^-3^ | 1.03 | T7 |
| DST**t**PAPTTKNNNNNNNNGGLVPRGSGGGSGHHHHHHHHHH | 12.64 | 4543.033 | 6 | -1.10 | 1.08 x 10^-1^ | 0.83 | T7 |
| MAPTTDS**tt**PAP**t**TKPTT | 13.27 | 2428.119 | 3 | 0.57 | 1.38 x 10^-3^ | 0.53 | T6, T7, T11 |
| TTDSTTPAPTTKPTTDST**t**PAP**tt**KP | 14.51 | 3225.527 | 4 | 0.11 | 5.43 x 10^-4^ | 2.32 | T7, T11, T12 |
| MAPTTDST**t**PAP**t**TKPTT | 14.78 | 2225.039 | 3 | 0.31 | 7.05 x 10^-3^ | 0.93 | T7, T11 |
| TTPAP**tt**KNNNNNNNNGGLVPRGSGGGSGHHHHHHHHHH | 16.14 | 4544.038 | 7 | -4.46 | 1.52 x 10^-2^ | 0.36 | T11, T12 |
| YTMAPT**t** | 16.42 | 987.434 | 2 | -0.51 | 1.38 x 10^-3^ | 1.17 | T3 |
| YTMAP**tt**DS**t**TPAPTTKPTT | 17.57 | 2692.227 | 4 | -0.54 | 1.38 x 10^-3^ | 0.46 | T2, T3, T6 |
| YTMAPTTDST**t**PAP**t**TKPTT | 18.90 | 2489.150 | 3 | 0.56 | 3.01 x 10^-2^ | 1.47 | T7, T11 |
| T**t**PAPTTKNNNNNNNNGGLVPRGSGGGSGHHHHHHHHHH | 19.84 | 4340.983 | 5 | 0.91 | 1.39 x 10^-3^ | 0.38 | T7 |
| MAPTTDSTTPAPT**t**KP**tt**D | 24.62 | 2543.152 | 5 | 3.15 | 4.12 x 10^-2^ | 0.38 | T2, T3, T12 |
